# Invasive *Enterobacter sakazakii* Disease in Infants

**DOI:** 10.3201/eid1208.051509

**Published:** 2006-08

**Authors:** Anna B. Bowen, Christopher R. Braden

**Affiliations:** * Centers for Disease Control and Prevention, Atlanta, Georgia, USA

**Keywords:** *Enterobacter sakazakii*, meningitis, infant, infant formula, research

## Abstract

*Enterobacter sakazakii* kills 40%–80% of infected infants and has been associated with powdered formula. We analyzed 46 cases of invasive infant *E. sakazakii* infection to define risk factors and guide prevention and treatment. Twelve infants had bacteremia, 33 had meningitis, and 1 had a urinary tract infection. Compared with infants with isolated bacteremia, infants with meningitis had greater birthweight (2,454 g vs. 850 g, p = 0.002) and gestational age (37 weeks vs. 27.8 weeks, p = 0.02), and infection developed at a younger age (6 days vs. 35 days, p<0.001). Among meningitis patients, 11 (33%) had seizures, 7 (21%) had brain abscess, and 14 (42%) died. Twenty-four (92%) of 26 infants with feeding patterns specified were fed powdered formula. Formula samples associated with 15 (68%) of 22 cases yielded *E. sakazakii*; in 13 cases, clinical and formula strains were indistinguishable. Further clarification of clinical risk factors and improved powdered formula safety is needed.

*Enterobacter sakazakii*, a gram-negative bacillus, is a rare cause of bloodstream and central nervous system infections (*1**–**8*). The organism has also been associated with necrotizing enterocolitis; however, it has not been firmly established as a causative agent (*8**–**10*). Reported outcomes are often severe: seizures; brain abscess; hydrocephalus; developmental delay; and death in as many as 40%–80% of cases (*11*). Premature infants are thought to be at greater risk than more mature infants, other children, or adults, and outbreaks of disease have occurred in hospital units for newborns (*1**,**2**,**4**,**6**,**8**–**10**,**12*). Infant infections with *E. sakazakii* have been associated with contaminated powdered formula products, but other environmental sources of contamination are possible (*1**,**3**,**9**,**10**,**13*). In this analysis, we attempt to more clearly define the host risk factors and disease course to refine prevention and treatment efforts.

## Methods

We reviewed the literature for reports of *E. sakazakii* disease in infants. Using medical subject heading terms "*E. sakazakii*" or "*Enterobacter*" in combination with "newborn," "infant," or "meningitis," we searched PubMed and examined the bibliographies of resulting articles. Finally, we reviewed *E. sakazakii* case consultations conducted by the Centers for Disease Control and Prevention (CDC) from 1998 to 2005 and reviewed results of a French outbreak reported to CDC in 2005 (*14*).

We defined a case as an infant (<12 months of age) with *E. sakazakii* cultured from an area of the body that is normally sterile: tissue, blood, cerebrospinal fluid, or urine aspirated through the bladder wall. Infants with cultures of cerebrospinal fluid or brain abscesses yielding *E. sakazakii* were considered to have meningitis. Because bacteremia is usually an intermediate step during the process of developing meningitis, patients with both bacteremia and meningitis were included in the meningitis group. Bacteremia was defined as *E. sakazakii* grown from the blood of a case-patient without evidence of meningitis.

Infants with gestational ages <37 weeks, or those reported to be premature, were considered premature. A gestational age of 40 weeks was assigned to 3 (27%) of 11 infants with bacteremia and 4 (14%) of 28 infants with meningitis who were reported as born following term gestation without a specific gestational age mentioned. We defined birthweight <2,500 g as low birthweight (LBW), <1,500 g as very low birthweight (VLBW), and <1,000 g as extremely low birthweight (ELBW).

Descriptive analysis was performed by using SAS software (SAS Institute, Cary, NC, USA). Groups were compared by using 2-tailed Fisher exact test.

## Results

Forty-six infants met the case definition (Table 1). Reported onset years ranged from 1958 to 2005; 32 (70%) cases were reported during the second half of this period. Cases were reported in 7 countries in North America, Europe, and the Middle East. Thirty-three (72%) infants had meningitis, 12 (26%) had bacteremia, and 1 (2%) had a urinary tract infection.

**Table 1:** Cases of invasive *Enterobacter sakazakii* disease among infants

Year of report	Location	No. cases	Reference
1961	England	2	(*15*)
1965	Denmark	1	(*5*)
1979	Georgia, USA	1	(*7*)
1981	Indiana, USA	1	(*16*)
	Oklahoma, USA	1	
1983	Netherlands	8	(*8*)
1985	Missouri, USA	1	(*17*)
1988	USA	2	
1989	Portugal	1	(*18*)
	Iceland	3	(*3*)
	Tennessee, USA	3	(*10*)
1990	Maryland, USA	1	(*19*)
1991	Ohio, USA	1	(*20*)
2000	North Carolina, USA	1	
2001	Israel	2	(*2**,**4*)
	Belgium	1	(*9*)
2002	Israel	2	(*4*)
	Tennessee, USA	1	(*1*)
	Wisconsin, USA	1	CDC, unpub. data
2003	USA	6	CDC, unpub. data
2004	France	2	(*14*)
	USA	2	CDC, unpub. data
2005	USA	2	CDC, unpub. data

Clinical characteristics were available for a subset of cases (Table 2). Eight (40%) of 20 infants for whom data were available were delivered by cesarean section. Twenty-nine (69%) of 42 infants experienced disease onset within a hospital. Gestational duration was available for 38 infants; 21 (55%) were born prematurely, and the median gestational age overall was 36 weeks (range 23.5–40 weeks). The median birthweight was 2,063 g (range 540–3,401); 18 (56%) of 32 infants had LBW; 9 (28%) of these met the definition for VLBW, and 7 (22%) met the definition for ELBW. Median age at the time of infection onset was 8.5 days (range 2–300).

**Table 2:** Characteristics of infants in the *Enterobacter sakazakii* case series

Characteristic	Published cases, n/N	Unpublished cases, n/N	Overall %
Male	15/30	6/9	54
Cesarean delivery	6/13	2/7	40
Nosocomial onset	24/31	5/11	69
Premature birth	17/29	4/9	55
Birthweight*			
LBW	16/27	2/5	56
VLBW	8/27	1/5	28
ELBW	6/27	1/5	22

*LBW, low birth weight; VLBW, very low birth weight; ELBW, extremely low birth weight.

Although the proportion of infants who experienced nosocomial disease onset was not significantly different between the meningitis and bacteremia groups, other infant characteristics differed by site of infection (Figure). The median gestational ages of infants with meningitis and bacteremia were 37 and 27.8 weeks, respectively (p = 0.02). Median birthweights were 2,454 g and 850 g, respectively (p = 0.002). However, median age at infection onset was 6 days in the group with meningitis and 35 days in the group with bacteremia (p < 0.0001). Thirty (94%) of 32 infants with meningitis, but only 2 (18%) of 11 infants with bacteremia, were <28 days old when infection was detected. One infant (8%) with bacteremia died; this infant also had necrotizing enterocolitis. The single infant with a urinary tract infection recovered without complication. In contrast, 14 (42%) of 33 infants with meningitis died. Of 19 surviving infants, only those with meningitis suffered adverse outcomes, including brain abscess (21%, p = 0.2), developmental delays (53%, p = 0.004), motor impairment (21%, p = 0.3), and ventricular shunt placement (42%, p = 0.01); 74% experienced at least one of these outcomes.

**Figure:**
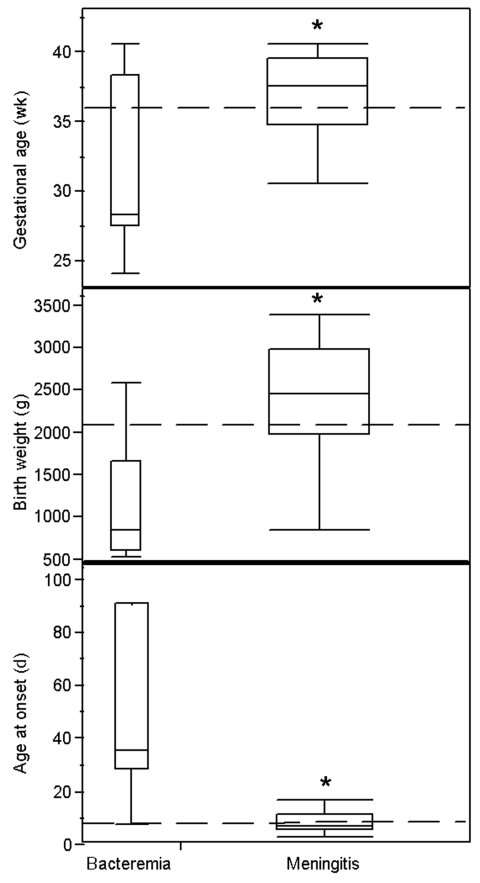
Box plot with each box indicating range (vertical lines), first and third quartiles (lower and upper boundaries of box, respectively), and median value (horizontal solid line) for gestational age in weeks, birth weight in grams, and age of onset in days for infants with bacteremia only or meningitis. The dotted lines indicate median values for all cases. *Significantly different values (α = 0.05) between groups.

Feeding practices were described for 26 infants. Twenty-four (92%) received a powdered formula product, including an infant who received powdered breast milk fortifier but no powdered infant formula; 1 additional infant received formula of an unspecified type. *E. sakazakii* was cultured from formula associated with 15 (68%) of 22 cases investigated. Isolates were obtained from prepared formula, opened formula tins, and previously unopened formula tins associated with 2, 6, and 7 cases, respectively. Thirteen (87%) of the 15 formula isolates were indistinguishable from the corresponding clinical strain by biotype or genotype; multiple formula manufacturers were implicated. In one of the remaining cases, multiple *E. sakazakii* strains were recovered from powdered formula, but none matched the clinical isolate by pulsed-field gel electrophoresis (CDC, unpub. data). In the other case, 2 *E. sakazakii* strains were isolated from blood and from rectal swabs; a third strain, as determined by arbitrarily primed PCR, was recovered from the powdered infant formula (*9*).

## Discussion

Although numerous reports include infants with *E. sakazakii* isolated from nonsterile sites, such as respiratory secretions or stool, 46 infants identified from the literature and CDC sources met the case definition for this analysis (*1**,**9**,**10**,**12*). Of the infants with sterile-site infection, 72% had meningitis. Contrary to previous characterizations of *E. sakazakii* disease, we found that infants with meningitis and bacteremia alone fell into 2 distinct groups. Those in whom meningitis developed tended to be of greater gestational age and birthweight than those with bacteremia alone. In fact, infants in whom meningitis developed tended to attain near-term gestational age and birthweight. In contrast, infants in whom bacteremia alone developed tended to be born very prematurely and have ELBW. A second major difference between the group with meningitis and the group with bacteremia was the infants' chronological ages. Infants with meningitis were generally <1 week of age at the onset of infection with *E. sakazakii*, whereas infants with bacteremia had generally surpassed the neonatal period at the onset of their disease. Rates of adverse outcome also differed between the 2 groups, although this was not unexpected. Most infants with *E. sakazakii* bacteremia fared better than those with meningitis. Among those in whom meningitis developed, rates of adverse outcome were similar to those reported in the literature: In this case series, 74% of meningitis survivors experienced an adverse neurologic outcome, while other studies cite adverse outcome in 20%–78% of neonatal or infant meningitis survivors (*21**–**23*).

The division of the infant population into 2 distinct groups occurred for unclear reasons. That infants <1 week of age comprised the meningitis group was not surprising; the disparities in other infant characteristics, however, are not intuitive. Since infants in the 2 groups experienced similar rates of nosocomial disease onset, the infants with bacteremia were unlikely to have simply received treatment earlier in the disease course than the infants with meningitis. Other risk factors are probable.

One likely set of risk factors is infant formula-feeding practices. Powdered infant formula is a demonstrated source of *E. sakazakii* infection. A microbiologic survey of powdered infant formulas published in 1988 found *E. sakazakii* in 20 (14%) of 141 samples tested (*24*). However, a survey of 82 powdered infant formula samples in 2003 yielded *E. sakazakii* in 2 (2.4%), which suggests that recent rates of powdered formula contamination may be lower (*25*). Powdered formula has also been implicated both epidemiologically and microbiologically as a vehicle in several cases of *E. sakazakii* disease in infants (*1**,**3**,**9**,**10**,**13*). Although we could not explore feeding exposures fully with the data available in this series, infant feeding practices may relate to the differences we describe in chronological age of infants at onset of meningitis and bacteremia. Infants with nearly normal gestational ages and birthweights are likely to be tended in normal newborn nurseries during the first 24–72 hours of life. In such nurseries, powdered formula is frequently given to babies who are not breast-fed, and it may also be used as a supplement by mothers who have chosen to breast-feed. Since infants are at highest risk for meningitis during the first several weeks of life, possibly because of immaturity of the blood-brain barrier, exposures to *E. sakazakii* in powdered formula or other sources during this time may quickly lead to central nervous system disease (*26**,**27*).

Conversely, in the intensive care settings where immature and low birthweight infants are tended, babies are not often fed powdered formula in the first few weeks of life. They may be given parenteral nutrition initially and may be fed breast milk from their own mothers or from a banked source when they do begin enteral feeds. If breast milk is not available, they are more likely to be given sterile, premixed infant formula than powdered formula, since standard preterm infant formula is only available in this form (*28*). Powdered breast milk fortifiers are not introduced until premature infants tolerate full-volume feeds, which may not occur for days or weeks after birth. Thus, infants in intensive care settings may not be exposed to nonsterile formula until they are more mature, which would lead to a greater proportion of *E. sakazakii* bacteremia than meningitis in this group if powdered formula is a source of infection. In this series, we were unable to explore the roles of indwelling enteric tubes, prior gastrointestinal surgery, and antecedent antacid or antimicrobial drug use as risk factors for infection.

While the reservoir for *E. sakazakii* is unknown, several environmental sources have been reported. *E. sakazakii* has been isolated from factories used to produce milk powder, chocolate, cereal, potato flour, spices, and pasta (*29*). It also has been isolated from household vacuum cleaner bags and from the guts of the stable fly, *Stomoxys calcitrans*, and the Mexican fruit fly, *Anastrpha ludens* (*29**–**31*). The relationship between these potential environmental sources and infant disease remains unclear. Although a human vaginal tract culture yielding *E. sakazakii* has been reported, vertical transmission is unlikely because nearly half of infants with *E. sakazakii* disease in this review were delivered by cesarean section, and symptoms developed in only 1 infant earlier than 3 days of age (*32*).

Our analyses were constrained by the use of retrospective and often incomplete data. Although cases with more severe outcomes might have been investigated and published more frequently than uncomplicated cases, this possible bias would not likely affect the representativeness of baseline infant characteristics. Assigning a gestational age of 40 weeks to term infants without a reported gestational age may have falsely elevated the median gestational ages we report, since most term infants are born at <40 weeks' gestation (*33*). However, a greater proportion of bacteremic infants than meningitic infants received this assignment, and therefore the significance of the differences in gestational age between the groups may be even greater than we report. We were unable to explore the effects of concomitant medical problems, treatments, and other environmental factors, and we relied on existing reports of feeding practices and formula testing. Clearly, additional study is needed to elucidate the lingering questions about *E. sakazakii* reservoirs, disease risk factors, and disease course.

Other gram-negative organisms, including *Escherichia coli*, *Enterobacter agglomerans*, *E. cloacae*, *Klebsiella pneumoniae*, *K. oxytoca*, and *Citrobacter freundii*, can be found in powdered infant formula (*24**,**25*). Powdered infant formula also has been associated with outbreaks of illness due to *Citrobacter* and multiple *Salmonella* serotypes (*13**,**34**–**38*). The degree to which *E. sakazakii* is a marker for a range of neonatal infections possibly related to powdered infant formula remains to be defined.

Certain steps can be taken immediately, however, to prevent or mitigate *E. sakazakii* disease. In a joint conference on infant formula safety in February, 2004, the World Health Organization and Food and Agriculture Organization of the United Nations made the following recommendations: 1) encourage industry partners to develop a range of affordable sterile formula options; 2) consider setting an industry standard for *Enterobacteriaecae* and *E. sakazakii* in infant formula; 3) inform infant caregivers of the risks associated with nonsterile, powdered formula; and 4) consider feeding high-risk infants sterile formula if they cannot breast-feed (*39*). The findings of our case review suggest that all neonates as well as premature infants should be included in this high-risk infant category. The American Dietetic Association has issued guidelines for infant formula preparation, storage, and administration; these should be followed by infant caregivers in hospitals and private homes (*40*). Rapid reporting of cases by clinicians could streamline data collection by local health departments and more rapidly resolve remaining questions about this illness. Manufacturer warning labels on powdered infant formula packages should stress that powdered infant formula is nonsterile and requires proper preparation, handling, and storage, and that sterile, liquid formula alternatives are available. These actions, adopted in whole or in part, may decrease the infectious risks associated with powdered formula and prevent this rare but potentially devastating disease.

## References

[1] Centers for Disease Control and Prevention. *Enterobacter sakazakii* infections associated with the use of powdered infant formula—Tennessee, 2001. MMWR Morb Mortal Wkly Rep. 2002;51:297–300. 12002167

[2] Bar-Oz B, Peleg O, Block C, Arad I. *Enterobacter sakazakii* infection in the newborn. Acta Paediatr. 2001;90:356–8. 10.1080/080352501300067857 11332182

[3] Biering G, Karlsson S, Clark N, Jonsdottir K, Ludvigsson P, Steingrimsson O. Three cases of neonatal meningitis caused by *Enterobacter sakazakii* in powdered milk. J Clin Microbiol. 1989;27:2054–6. 2778070 10.1128/jcm.27.9.2054-2056.1989 PMC267737

[4] Block C, Peleg O, Minster N, Bar-Oz B, Simhon A, Arad I, Cluster of neonatal infections in Jerusalem due to unusual biochemical variant of *Enterobacter sakazakii.* Eur J Clin Microbiol Infect Dis. 2002;21:613–6. 10.1007/s10096-002-0774-5 12226694

[5] Joker RN, Norholm T, Siboni KF. A case of neonatal meningitis caused by a yellow *Enterobacter.* Dan Med Bull. 1965;12:128–30. 5825012

[6] Lai KK. *Enterobacter sakazakii* infections among neonates, children, and adults. Medicine. 2001;80:113–22. 10.1097/00005792-200103000-00004 11307587

[7] Monroe PW, Tift WL. Bacteremia associated with *Enterobacter sakazakii* (yellow pigmented *Enterobacter cloacae*). J Clin Microbiol. 1979;10:850–1. 521484 10.1128/jcm.10.6.850-851.1979 PMC273282

[8] Muytjens HL, Zanen H, Sonderkamp H, Kollee L, Wachsmuth I, Farmer J. Analysis of eight cases of neonatal meningitis and sepsis due to *Enterobacter sakazakii.* J Clin Microbiol. 1983;18:115–20. 6885983 10.1128/jcm.18.1.115-120.1983 PMC270753

[9] Van Acker J, de Smet F, Muyldermans G, Bougatef A, Naessens A, Lauwers S. Outbreak of necrotizing enterocolitis associated with *Enterobacter sakazakii* in powdered milk formula. J Clin Microbiol. 2001;39:293–7. 10.1128/JCM.39.1.293-297.2001 11136786 PMC87717

[10] Simmons BP, Gelfand MS, Haas M, Metts L, Ferguson J. *Enterobacter sakazakii* infections in neonates associated with intrinsic contamination of a powdered milk formula. Infect Control Hosp Epidemiol. 1989;10:398–401. 10.1086/646060 2794464

[11] Nazarowec-White M, Farber J. *Enterobacter sakazakii*: a review. Int J Food Microbiol. 1997;34:103–13. 10.1016/S0168-1605(96)01172-5 9039558

[12] Arseni A, Malamou-Ladas E, Koutsia C, Xanthou M, Trikka E. Outbreak of colonization of neonates with *Enterobacter sakazakii.* J Hosp Infect. 1987;9:143–50. 10.1016/0195-6701(87)90052-1 2883221

[13] Centers for Disease Control and Prevention. *Salmonella* serotype Tennessee in powdered milk products and infant formula–Canada and United States, 1993. MMWR Morb Mortal Wkly Rep. 1993;42:516–7. 8515742

[14] Coignard B, Vaillant V, Vincent JP, Leflèche A, Mariani-Kurkdjian P, Bernet C, Infections sévères à *Enterobacter sakazakii* chez des nouveau-nés ayant consommé une préparation en poudre pour nourrissons, France, octobre-décembre 2004. Available from: http://www.invs.sante.fr/BEh/2006/02_03/beh_02_03_2006.pdf

[15] Urmenyi AM, Franklin AW. Neonatal death from pigmented coliform infection. Lancet. 1961;1:313–5. 10.1016/S0140-6736(61)91481-7 13779326

[16] Kleiman MB, Allen SD, Neal P, Reynolds J. Meningoencephalitis and compartmentalization of the cerebral ventricles caused by *Enterobacter sakazakii.* J Clin Microbiol. 1981;14:352–4. 7287892 10.1128/jcm.14.3.352-354.1981 PMC271970

[17] Naqvi SH, Maxwell MA, Dunkle LM. Cefotaxime therapy of neonatal gram-negative bacillary meningitis. Pediatr Infect Dis. 1985;4:499–502. 10.1097/00006454-198509000-00012 3900946

[18] Lecour H, Seara J, Miranda M. Treatment of childhood bacterial meningitis. Infection. 1989;17:343–6. 10.1007/BF01650726 2689353

[19] Noriega FR, Kotloff KL, Martin MA, Schwalbe RS. Nosocomial bacteremia caused by *Enterobacter sakazakii* and *Leuconostoc mesenteroides* resulting from extrinsic contamination of infant formula. Pediatr Infect Dis J. 1990;9:447–9. 10.1097/00006454-199006000-00018 2114609

[20] Gallagher PG, Ball WS. Cerebral infarctions due to CNS infection with *Enterobacter sakazakii.* Pediatr Radiol. 1991;21:135–6. 10.1007/BF02015629 2027718

[21] Stevens JP, Eames M, Kent A, Halket S, Holt D, Harvey D. Long term outcome of neonatal meningitis. Arch Dis Child Fetal Neonatal Ed. 2003;88:F179–84. 10.1136/fn.88.3.F179 12719389 PMC1721546

[22] Bedford H, de Louvais J, Halket S, Peckham C, Hurley R, Harvey D. Meningitis in infancy in England and Wales: follow up at age 5 years. BMJ. 2001;323:533–6. 10.1136/bmj.323.7312.533 11546697 PMC48156

[23] Harvey D, Holt D, Bedford H. Bacterial meningitis in the newborn: a prospective study of morbidity and mortality. Semin Perinatol. 1999;23:218–25. 10.1016/S0146-0005(99)80066-4 10405191

[24] Muytjens HL, Roelofs-Willemse H, Jaspar GH. Quality of powdered substitutes for breast milk with regard to members of the family *Enterobacteriaceae.* J Clin Microbiol. 1988;26:743–6. 3284901 10.1128/jcm.26.4.743-746.1988 PMC266435

[25] Iversen C, Forsythe S. Isolation of *Enterobacter sakazakii* and other *Enterobacteriaceae* from powdered infant formula milk and related products. Food Microbiol. 2004;21:771–7. 10.1016/j.fm.2004.01.009

[26] Schuchat A, Robinson K, Wenger J, Harrison L, Farley M, Reingold A, Bacterial meningitis in the United States in 1995. N Engl J Med. 1997;337:970–6. 10.1056/NEJM199710023371404 9395430

[27] Wenger JD, Hightower AW, Facklam RR, Gaventa S, Broome CV. Bacterial meningitis in the United States, 1986: report of a multistate surveillance study. J Infect Dis. 1990;162:1316–23. 10.1093/infdis/162.6.1316 2230261

[28] Current marketing and use of powdered infant formula in the United States, 2003, US Food and Drug Administration. Available from http://www.fda.gov/ohrms/dockets/ac/03/briefing/3939b1_tab4c_coversheet.pdf

[29] Kandhai MC, Reij MW, Gorris LG, Guillaume-Gentil O, van Schothorst M. Occurrence of *Enterobacter sakazakii* in food production environments and households. Lancet. 2004;363:39–40. 10.1016/S0140-6736(03)15169-0 14723994

[30] Hamilton JV, Lehane MD, Braig HR. Isolation of *Enterobacter sakazakii* from midgut of *Stomoxys calcitrans.* Emerg Infect Dis. 2003;9:1355–6. 14626227 10.3201/eid0910.030218 PMC3033080

[31] Kuzina LV, Peloquin JJ, Vacek DC, Miller TA. Isolation and identification of bacteria associated with adult laboratory Mexican fruit flies, *Anastrepha ludens.* Curr Microbiol. 2001;42:290–4. 10.1007/s002840110219 11178731

[32] Ongradi J. Medline Vaginal infection by *Enterobacter sakazakii.* Sex Transm Infect. 2002;78:467–8. 10.1136/sti.78.6.467-a 12473818 PMC1758338

[33] Martin JA, Hamilton BE, Sutton PD, Ventura SJ, Menacker F, Munson ML. Births: final data for 2002. Natl Vital Stat Rep. 2003;52:1–113. 14717305

[34] Collins RN, Treger MD, Goldsby JB, Boring JR, Coohon DB, Barr RN. Interstate outbreak of *Salmonella newbrunswick* infection traced to powdered milk. JAMA. 1968;203:838–44. 10.1001/jama.203.10.838 5694205

[35] Thurm V, Gericke B. Identification of infant food as a vehicle in a nosocomial outbreak of *Citrobacter freundii*: epidemiological subtyping by allozyme, whole-cell protein and antibiotic resistance. J Appl Bacteriol. 1994;76:553–8. 10.1111/j.1365-2672.1994.tb01652.x 8027004

[36] Usera MA, Echeita A, Aladuena A, Blanco MC, Reymundo R, Prieto MI, Interregional foodborne salmonellosis outbreak due to powdered infant formula contaminated with lactose-fermenting *Salmonella virchow.* Eur J Epidemiol. 1996;12:377–81. 10.1007/BF00145301 8891542

[37] Threlfall EJ, Ward LR, Hampton MD, Ridley AM, Rowe B, Roberts D, Molecular fingerprinting defines a strain of *Salmonella enterica* serotype anatum responsible for an international outbreak associated with formula-dried milk. Epidemiol Infect. 1998;121:289–93. 10.1017/S0950268898001149 9825779 PMC2809525

[38] Rowe B, Begg N, Hutchinson D, Dawkins H, Gilbert R, Jacob M, *Salmonella Ealing* infections associated with consumption of infant dried milk. Lancet. 1987;2:900–3. 10.1016/S0140-6736(87)91384-5 2889093

[39] *Enterobacter sakazakii* and other microorganisms in powdered infant formula: meeting report. In: Microbiological risk assessment series, no. 6.Geneva: World Health Organization; 2004.

[40] American Dietetic Association. Infant feedings: guidelines for preparation of formula and breast milk in health care facilities, 2003, Washington: American Dietetic Association; 2004.

